# Duration of farming is an indicator of natural restoration potential of sedge meadows

**DOI:** 10.1038/s41598-017-11429-0

**Published:** 2017-09-06

**Authors:** Guodong Wang, Ming Wang, Xianguo Lu, Ming Jiang

**Affiliations:** 1 Key Laboratory of Wetland Ecology and Environment, Northeast Institute of Geography and Agroecology, Chinese Academy of Sciences, Changchun, Jilin, 130102 China; 2 Institute for Peat and Mire Research, Northeast Normal University, Changchun, Jilin, 130024 China

## Abstract

Soil seed banks can be important components of ecological restoration, particularly if the species remain viable in the soil for long periods of time. A germination experiment was conducted in the greenhouse to determine seed bank viability based on length of time farmed. Soils from sedge meadows farmed between 0 and 50 years were collected in Sanjiang Plain, China. Most dominant sedges (e.g., *Carex schmidtii*, *C. lasiocarpa*) and grasses (e.g. *Calamagrostis angustifolia*) survived as seeds if farmed for less than 5 years, therefore fields farmed for short periods of time are the best candidates for wetland restoration. Certain important structural components (tussock-forming *Carex* spp.) are not retained in seed banks when farmed for 6–15 years, but the seed banks still contained viable seeds of other important sedge meadow species, which could contribute to the restoration of wetland communities. However, most sedge meadow species were missing in fields farmed for more than 16 years, which make these fields difficult to restore via natural recolonization. We conclude that the duration of farming can be used as a general indicator of the potential of natural restoration for sedge meadows. This information could be used to determine which wetlands might be targeted for restoration.

## Introduction

Vast tracts of the wettest agricultural fields have been abandoned world-wide, and an understanding of their restoration potential as nature conservation areas is becoming increasingly important^1^. Since 1950, 80% of sedge meadows in the Sanjiang Plain, northeast of China, have been converted to agriculture^2^. Similarly in Europe, more than 50% of original wetlands have been lost in France, Germany, Greece, Italy, the Netherlands and Spain, mostly due to land drainage and agricultural intensification^3^. Some of these converted floodplains are no longer farmed, in part because they may be unsuitable for cultivation but partly also due to the recognition of the national and global environmental significance of wetlands and of the rich biodiversity that they support^4, 5^. In the Heilongjiang Province, northeast of China, 42,000 ha of these floodplains will be restored from 2017 to 2020^6^. The study of seed availability from remnant seed banks is particularly relevant because of the need to better understand the restoration potential of sedge meadows converted to farm fields.

Whether or not species will naturally re-establish in formerly farmed sedge meadows depends to a large extent on the availability of seeds in the seed bank^7, 8^. Soil seed banks provide seeds for the regeneration of plant communities, and can be of value in the natural restoration of farm fields to wetlands if the seeds have survived cultivation^9, 10^. If the seeds of key structural dominants are absent from seed banks, farm fields can be difficult to restore to original wetlands^11, 12^. Interest in seed banks for aiding the rehabilitation and reconstruction of damaged sedge meadows is growing^13, 14^. However, European and North American studies suggest that important components of vegetation are often missing from naturally restored wetlands^11, 15^. In the Prairie Pothole Region, where wetlands were restored via natural recolonization, more than 60 *Carex* species, some of which are key structural components of tussock meadows, rarely return after restoration^16^. Similar conditions were also found in Europe^15, 17^. The value of the seed bank to restoration varies greatly according to wetland type, the duration of the farming, and the longevity of the seeds^7, 9, 10, 18^.

Long-lived seeds could remain viable in the soil for long periods of time so it is possible for these species to regenerate after long-term farming, while short-lived seeds more readily disappear from seed banks^1^. Seeds of many sedges are long-lived, so they might be able to remain viable in the seed bank of sedge meadows for extended periods, but for other species viability may be lost in less than 3 years^19^. Schütz (2000) reported that the longevity for 10 *Carex* species could be estimated from seed bank studies and was at least 10 years^20^, while van der Valk *et al*. (1999) found that the viability of three *Carex* species dropped to less than 7% after only one year^21^.

However, because studies related to the impact of various durations of cultivation on seed banks are generally lacking, little is known about whether a time threshold exists that defines the boundary between the possibility of natural recovery and the need for artificial restoration of sedge meadows converted to farm fields. The objectives of this study were to examine whether the duration of farming could be used as an indicator of the natural restoration potential of sedge meadows converted to farm fields in Sanjiang Plain, northeast of China. If important components are missing from the seed bank, then it is unlikely that sedge meadows will spontaneously develop. The species richness and seed density of seed banks in sedge meadows, which were either intact or farmed between 1 and 50 years, were examined. We hypothesized that the duration of farming could be used as a general indicator of the natural restoration potential of sedge meadows converted to farm fields. Specifically, we hypothesized that the species richness and density of seeds in the seed bank would decrease as the length of time of farming increased.

## Results

### Composition and Density of Soil Seed Banks of Natural Wetlands and Farm Fields

A total of 110 species germinated from the soil, including 94 wetland species and 16 non-wetland species (Supplementary Table S1). There were 97 species in the natural wetlands, and 79 in the farm fields (Supplementary Table S1). Ninety-two species germinated from natural wetlands under the drained condition. Dominant species included the perennial species *Calamagrostis angustifolia*, *Glyceria spiculosa*, *Juncus papillosus*, *Polygonum amphibium* and the annual species *Gnaphalium mandshuricum*. Tussock-forming species in the seed banks included *Carex appendiculata*, *C. meyeriana* and *C. schmidtii*. Woody species included *Salix myrtilloides*, *S. rosmarinifolia*, *S. floderusii* and *Spiraea salicifolia*. Ten species germinated from natural wetlands under the flooded condition, including submerged species such as *Ceratophyllum demersum*, *Potamogeton crispus*, *P. malaianus*, *P. perfoliatus*, *Vallisneria spiralis* and emergent species such as *Alisma orientale*, *Sagittaria trifolia*, *Sium suave*, *Typha angustifolia* (Supplementary Table S1).

Seventy-six species germinated from farm fields under drained conditions (Supplementary Table S1). Dominant species included perennial species *Calamagrostis angustifolia*, *Rorippa palustris*, *Polygonum amphibium*, and annual species *Gnaphalium mandshuricum* and *Chenopodium glaucum*. Seven species germinated from farm fields under the flooded condition, including *A. orientale*, *Callitriche palustris*, *P. crispus*, *P. malaianus*, *S. trifolia*, *T. angustifolia* and *V. spiralis* (Supplementary Table S1).

**Figure 1:**
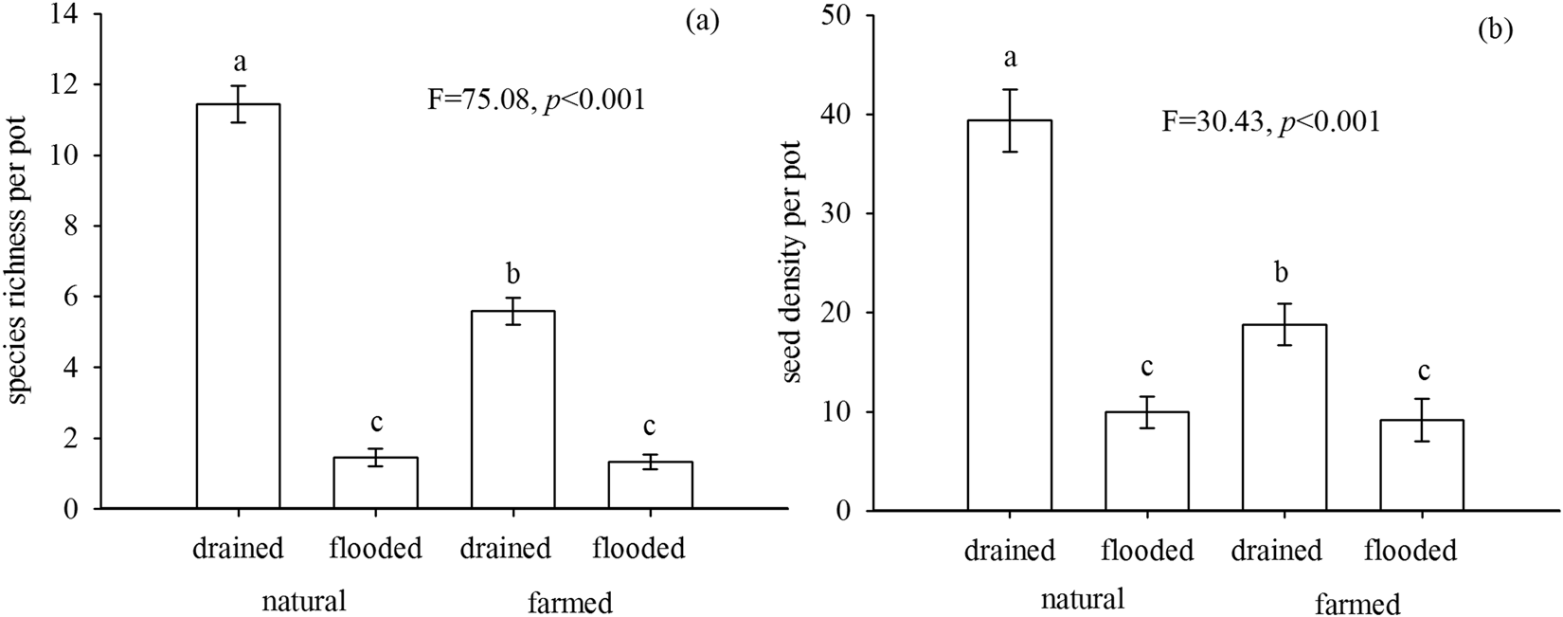
Significant interactions of site type and water regime for a greenhouse seed bank assay from soil collected in the Sangjiang Plain, northeastern China, including (**a**) species richness per pot, and, (**b**) seed density per pot. Site types included natural and farmed sedge meadows; water regime treatments in the greenhouse study included drained and flooded. Different letters indicate significant differences in means (*p* < 0.05).

Species richness and seed density per pot were significantly higher in the drained condition than in the flooded condition both in wetlands and farm fields (Fig. 1). In the drained water treatment, species richness and seed density per pot were higher in natural wetlands than in farm fields (Fig. 1), but there was no significant difference between natural wetland and farm fields in the flooded water treatment (Fig. 1).

### Soil Seed Bank Changes over Duration of Farming

**Figure 2:**
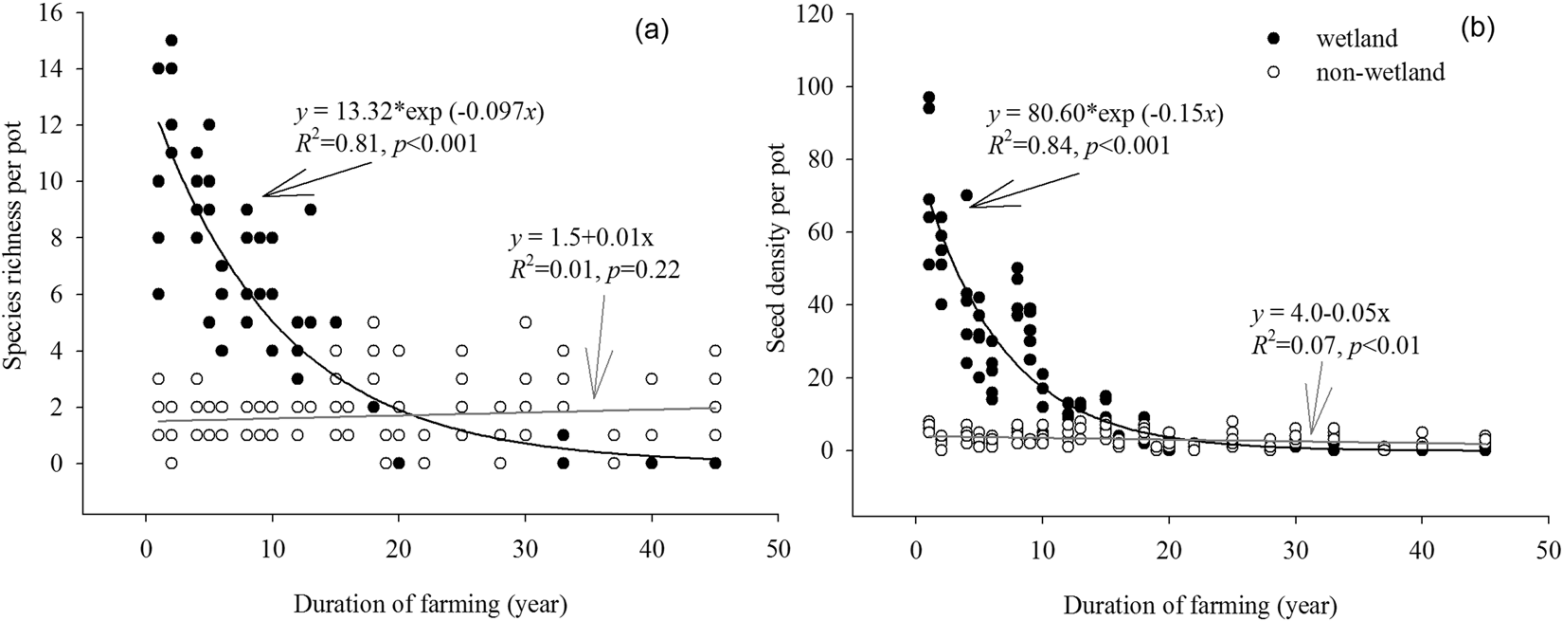
The relationship between species richness (**a**)/seed density (**b**) of the wetland and non-wetland categories per pot per site and the duration of farming for the soybean fields in Sanjiang Plain, northeastern China. Exponential and linear regressions were fit for values of wetland and non-wetland species, respectively.

There were significantly negative correlations between the richness of wetland species and the duration of farming in the soybean fields. The duration of farming explained 81% of the richness variation based on an exponential regression model (y = ae^bx^, *p* < 0.001; Fig. 2a). The richness of non-wetland species was low in soybean fields farmed for any length of time (0–5 species; Fig. 2a).

Patterns of seed density were consistent with species richness throughout the study. The seed density of wetland species decreased as the duration of farming increased, and explained 84% of the seed density variation based on an exponential regression model (Fig. 2b). The seed density of non-wetland species was low in soybean fields farmed for any length of time (<15 seeds; Fig. 2b).

**Figure 3:**
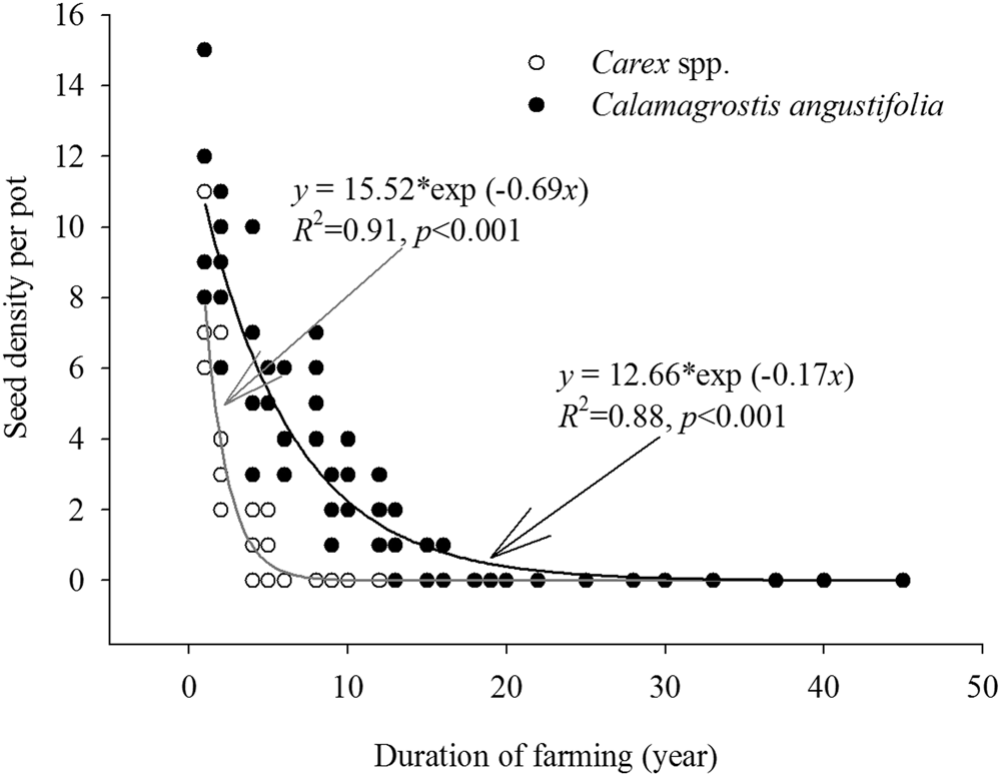
The relationship between the seed density of the dominant sedges (*Carex* spp.) and grass (*Calamagrostis angustifolia*) per pot per site and the duration of farming for the soybean fields in Sanjiang Plain, northeastern China. Exponential regressions were fit to the data.

As the dominant species of sedge meadows, the seed density of the *Carex* spp. as well as *Calamagrostis angustifolia* decreased over time (Fig. 3). Seed density of *Carex* spp. decreased significantly with time of farming, with a small number of seeds retained in soybean fields farmed for less than 5 years (Fig. 3). Seed density of *C. angustifolia* decreased in soybean fields farmed from 1 to 15 years, but were absent in field farmed for more than 15 years (Fig. 3).

## Discussion

Farmland abandonment is increasing around the world, and provides an opportunity to restore wetlands^7^. In some parts of the world, however, it has proved difficult to restore the full complement of plant species through natural regeneration^7, 15, 22^. Similarly, restoration by replanting has often resulted in lack of foundation species or low species richness^23^. Better methods of restoring these important ecosystems are now required and abandoned farm fields that were originally sedge meadows provide an opportunity to investigate alternative approaches to restoration. This study examined the composition of seed banks of soybean fields farmed between 1 and 50 years to determine the utility of soil seed banks for the restoration of sedge meadows.

**Figure 4:**
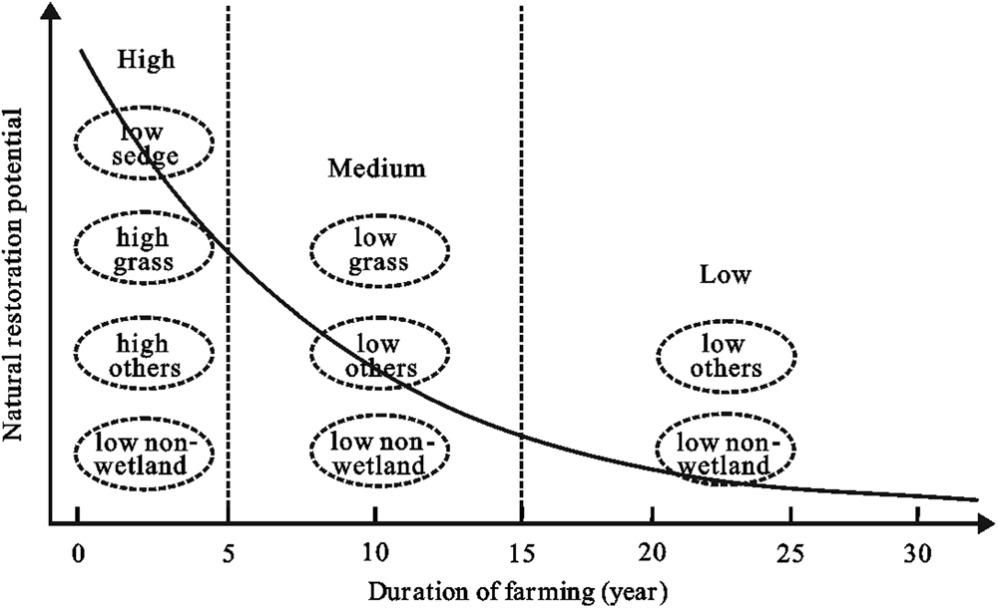
A hypothetical conceptual model outlining the relationships between the duration of farming and the natural restoration potential of sedge meadows. Solid curve represents the response of natural restoration potential to the duration of farming. Period 1 (0–5 years), period 2 (6–15 years), and period 3 (>16 years) represent high, medium, and low natural restoration potential ranges. Dashed circles show the seed density of different categories (wetland species (sedge, grass and others) and non-wetland species).

The loss of native species from soil seed banks was related to the duration of farming in our study, as shown in a conceptual model (Fig. 4). The relationship of duration of farming to restoration potential can be categorized as follows: high (0–5 years), medium (6–15 years), and low (>16 years). During the high restoration potential period, a high density of seeds of certain sedge meadow species germinated such as *Calamagrostis angustifolia*, *Salix rosmarinifolia*, *Polygonum persicaria*, *Galium trifidum* and *Comarum palustre*. Dominant *Carex* species such as *Carex lasiocarpa*, *C. limosa*, *C. humida*, *C. pseudo-curaica* and *C. orthostachys* were also maintained in the seed banks although the seed density was relatively low. In the medium restoration potential period (6–15 years), grasses (e.g., *C. angustifolia*) and other wetland species (e.g., forbs, shrubs) were common in the soil seed banks, but the seed density was much lower than that in the natural sedge meadows. *Carex* species disappeared in the fields farmed for 6–15 years. Our findings are consistent with other studies. For example, 6 years after farm abandonment and natural restoration (i.e., no replanting), dominant tussock *Carex* species (*C. appendiculata* and *C. schmidtii*) and many other sedge meadow species successfully recolonized the Qixing restoration site, which had been farmed for only 3 years prior to restoration in Naoli River National Natural Reserve, Sanjiang Plain, China (4,000 ha restoration site)^24^. In two restored sites that were farmed for 7 and 14 years before restoration, the two restored sedge meadows were comprised of vegetation such as *C. angustifolia*, and shrubs such as *S. rosmarinifolia*, *S. myrtilloides*, and *S. salicifolia*. But the dominant tussock sedge species *C. meyeriana* and *C. appendiculata* did not germinate in the two restored sedge meadows even after about 10 years’ natural restoration^12^. Similarly, in a restoration site in Wicken Fen National Nature Reserve in Cambridgeshire, U.K., while seed banks contributed to non-tussock wetland communities, *Carex* spp. did not recolonize even decades after restoration, despite their proximity to species-rich habitat^15^. In fields cultivated for more than 15 years (low restoration potential), most of the wetland species present in the seed banks of natural wetlands were absent, and the non-wetland species (e.g., *Chenopodium glaucum*) dominated the seed bank. A case study in one restoration site farmed for 24 years in Sanjiang Plain found that numerous species common to natural sedge-dominated meadow were notably absent after three years’ natural recolonization, and *C. glaucum* became the most dominant species in the site (660 ha restoration site; Hongwei County, Heilongjiang Province, China; C. Ding, August 2015, Naoli River National Natural Reserve, personal communication). Similarly, a study in a prairie potholes wetland indicated that most of wetland species were lost if farming continued for more than 20 years^18^. Similarly, sedge meadow species including *Calamagrostis canadensis*, *Carex lacustris* and *Lysimachia thrysiflora* failed to recolonize in farmland farmed for decades via natural recolonization in prairie pothole wetlands^22^.

Overall, the duration of farming significantly affected the seed bank viability and the wetland restoration potential of natural sedge meadows converted to farm fields. In the high restoration potential period with less than 5 years of cultivation, most of the sedge meadow species including certain dominant *Carex* spp. were still maintained in the seed bank, these fields were the best candidates for wetland restoration. In the medium restoration potential period (6–15 years), although certain critical components of the vegetation were not retained in seed banks, the seed banks still contained viable seeds of many wetland species and so could contribute toward the restoration of novel wetland vegetation assemblages. In contrast, fields farmed for more than 16 years had lost most of the sedge meadow species from the seed banks. Our findings indicate that the duration of farming could be an indicator of the natural restoration potential of sedge meadows.

Beyond the seed bank composition, the hydrology of a restored field plays an integral role in seed dispersal, seed bank recruitment, and vegetation establishment^7, 25^. Water is the environment filter that regulates the germination of the seed bank^1, 26^, and the germination of wetland species of various life history types require either drawdown or flooding^27^. In this study, species richness was high and emergent plants (e.g., *Calamagrostis*, *Carex*) dominated in the seed bank under the drained water regime, while emergent or submerged plants (e.g., *Typha*, *Potamogeton*, *Sagittaria*) dominated in the seed bank under continuous flooding. Thus, from the perspective of restoration, the species richness of seeds in farm fields may be adequate to restore many sedge meadow species provided the hydrology includes alternating flooding and drawdown periods to maximize the germination of species of various types. In addition, *T. angustifolia* dominated the seed bank in farm fields under continuous flooded water regime (Supplementary Table S1). Similarly, changes in water levels, which promote *Typha* spp. have enhanced the establishment and persistence of invasive cattail in Great lakes and other regions^28^. In Fujin National Wetland Park in Sanjiang Plain, large tracts of farmed sedge meadows have been restored using natural recolonization since 2009. Unfortunately, after initial reflooding (>1 m water depth), non-target *T*. *angustifolia* efficiently recolonized and sedge meadow species did not^29^. Cattail invasion is often correlated with reduced hydrologic variability or otherwise altered hydrology as well as elevated nutrients^30, 31^. Once established, the species tends to persist by tolerating a wide range of water level conditions^32^, form dense, nearly monotypic stands and resists the establishment of a native plant community^33^. Thus, the success or failure of a project aimed at restoring the biodiversity of farm fields depends on water regimes, as well as the seed bank.

## Methods

### Study Sites

The Sanjiang Plain wetlands, located in the northeastern Heilongjiang Province, comprise one of the richest areas of globally significant biodiversity in the People’s Republic of China. Sedge meadows are located at the confluence of the Songhua, Heilongjiang, and Wusuli Rivers and along the floodplains of these rivers. For this study, sedge meadows were selected along the Nongjiang River, which is 14.8% of the Sanjiang Plain watershed. In 1954, wetlands covered more than 72% of this area. Since then wetlands have been reclaimed mostly for soybean cultivation. By 2005, soybean fields covered 764,391 ha of the region, and wetlands covered less than 12%^34^.

**Figure 5:**
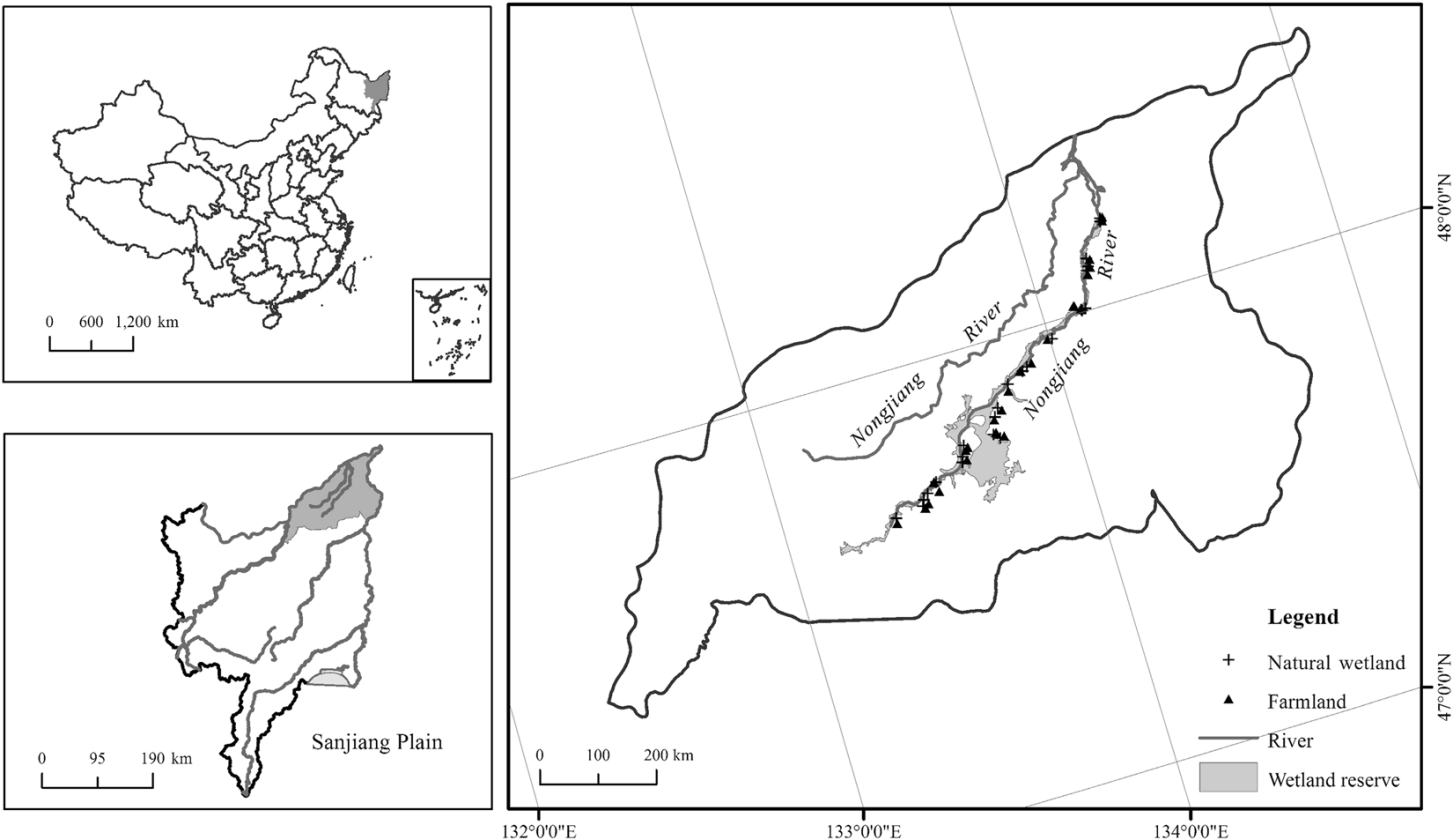
Site locations for seed bank samples locations along the Nongjiang River, Sanjiang Plain, China. The maps were generated by Ming Wang using ArcGIS 10.0.

For this study, the seed banks of soybean fields and intact sedge meadows were selected throughout the floodplain of the Nongjiang River (Fig. 5), in Heilongjiang Province near the southern edge of the sub-arctic zone. We sampled soybean fields, which had been converted from sedge meadows for various periods of time (1–50 years). Soybean fields are usually tilled at the end of May and harvested in October. Along with these farm fields, adjacent sedge meadows that were still intact also were sampled. Dominant sedge meadow species include sedges such as *Carex meyeriana*, *C. lasiocarpa*, *C. schmidtii*, grasses such as *Calamagrostis angustifolia*, and various forbs and shrubs. Tussock-forming sedges include *Carex appendiculata*, *C. meyeriana* and *C. schmidtii* ^35^.

### Seed Bank Sampling

Sampling was conducted right after snow melt (May 2–20, 2015) to assess the seed bank present at the time of spring germination including both transient and persistent types of seeds. In soybean fields converted from sedge meadows, we chose twenty-three sites with different farming durations as determined by talking with local landowners. Twenty-three natural sedge meadows, which had never been farmed were sampled adjacent to these farm fields along Nongjiang River. At each site, five quadrats (25 × 25 × 10 cm), which were at least 10 m apart from each other were chosen randomly, and soil samples were placed in gallon sized plastic bags. A total of 230 soil samples were collected (46 sites × 5 samples/site).

### Site Environment and Vegetation Characteristics

The study area has a temperate continental monsoon climate with a mean annual (1990–2010) temperature of 2.53 °C (monthly range of average temperature of −20.4 to 21.6 °C), a mean annual precipitation of 566 mm (approximately 60% fall in July and August) and a frost-free period of approximately 125 days per year. Longitude and latitude of all sites were recorded using a GPS. Water depths were estimated with a measuring stick at time of collection, and the duration of farming was recorded for each site. Dominant species and subdominant species of standing field vegetation of seed bank sampling areas in natural wetlands were recorded.

### Experimental Design

Soil samples were placed in an unheated greenhouse until the experiment began (May 21–24, 2015). Seedling germination assays were carried out in the greenhouse. Soil samples were sieved with water to push samples through the screen and remove debris and roots. After sieving to remove roots, each soil sample was mixed well. Soil samples for each site were divided into 10 subsamples to create 5 replicates for each of the two water treatments. Each soil sample was spread as an even layer, 2 cm thick, in pots (25 × 25 cm) previously filled with washed vermiculite to 8 cm depth. Trays were placed in tanks assigned to one of two water treatments either drained (moist soil) or flooded (10 cm depth). The trays were watered by filling the tanks from the bottom. Newly emerged seedlings were identified, counted, and removed from the pots. If a seedling could not be identified, it was removed from the original pot and grown in a separate pot until it could be identified. Seed germination assays continued for six months. Nomenclature follows Yi *et al*. (2008)^35^ and Fu (1995)^36^.

### Data analysis

Mean seed densities for each species were calculated. Two-way analysis of variance (ANOVA) including site type, water regime and their interactions was conducted for species richness and seed density. Tukey’s comparisons were performed to compare means in situations with significant interactions of main effects. Significance was determined at an alpha level of 0.05. Data were appropriately transformed to satisfy the assumptions of ANOVA.

Species were divided into wetland and non-wetland categories^35^. Species richness and seed density of the wetland and non-wetland categories for the soybean fields were regressed over duration of farming using linear, quadratic, inverse, logistic, logarithmic and exponential models respectively. We finally found that exponential regression analysis could best explain the variability in the data of wetland category. At the same time, we used linear regression analysis to explain the variability in the data of non-wetland category. In order to further explain the restoration potential of farmed sedge meadows, the seed density of the dominant sedges (*Carex* spp.) and grass (*Calamagrostis angustifolia*) respectively were regressed over duration of farming using exponential models. All statistics were conducted using SPSS version 16.0.

### Data availability

The datasets generated during and/or analysed during the current study are available from the corresponding author on reasonable request.

## Electronic supplementary material

Supplementary Table S1

## References

[1] Middleton, B. A. *Wetland restoration, flood pulsing and disturbance dynamics*. John Wiley and Sons, New York (1999).

[2] Wang ZM (2011). Loss and Fragmentation of Marshes in the Sanjiang Plain, Northeast China, 1954-2005. Wetlands.

[3] Jones, T. A. & Hughes, J. M. R. Wetland inventories and wetland loss studies: a European perspective. *Waterfowl and wetland conservation in the 1990s: A global perspective*, 164–169 (1993).

[4] Newling CJ (1990). Restoration of bottomland hardwood forests in the Lower Mississippi Valley. Ecol. Restor..

[5] Berdach, J. T. *et al*. *Reviving Lakes and Wetlands in the People’s Republic of China, Volume 3: Best Practices and Prospects for the Sanjiang Plain Wetlands*. http://hdl.handle.net/11540/6628 (2016).

[6] Cui XB, Liu MH, Ma YK (2016). Benefit analysis for converting croplands to wetlands project in Naoli river national nature reserve of Heilongjiang. Heilongjiang Sci..

[7] Middleton BA (2003). Soil seed banks and the potential restoration of forested wetlands after farming. J. Appl. Ecol..

[8] Merritt DJ, Dixon KW (2011). Restoration seed banks-a matter of scale. Science.

[9] van der Valk AG, Pederson RL, Davis CB (1992). Restoration and creation of freshwater wetlands using seed banks. Wetl. Ecol. Manag..

[10] Wang GD, Wang M, Lu XG, Jiang M (2015). Effects of farming on the soil seed banks and wetland restoration potential in Sanjiang Plain, Northeastern China. Ecol. Eng..

[11] Kettenring KM, Galatowitsch SM (2011). *Carex* seedling emergence in restored and natural prairie wetlands. Wetlands.

[12] Wang GD, Middleton B, Jiang M (2013). Restoration potential of sedge meadows in hand-cultivated soybean fields in Northeastern China. Restor. Ecol..

[13] Hong J, Liu S, Shi G, Zhang Y (2012). Soil seed bank techniques for restoring wetland vegetation diversity in Yeyahu Wetland, Beijing. Ecol. Eng..

[14] Wang M, Wang GD, Lu XG, Jiang M, Wang SZ (2016). Soil seed banks and their implications for wetland restoration along the Nongjiang River, Northeastern China. Ecol. Eng..

[15] Stroh PA, Hughes FMR, Sparks TH, Mountford JO (2012). The influence of time on the soil seed bank and vegetation across a landscape-scale wetland restoration project. Restor. Ecol..

[16] Aronson MFJ, Galatowitsch SM (2008). Long-term vegetation development of restored prairie pothole wetlands. Wetlands.

[17] Blomqvist MM, Bekker RM, Vos P (2003). Restoration of plant species richness: the potential of the soil seed bank. Appl. Veg. Sci..

[18] Wienhold CE, van der Valk AG (1989). The impact of duration of drainage on the seed banks of northern prairie wetlands. Can. J. Bot..

[19] Leck MA, Schütz W (2005). Regeneration of *Cyperaceae*, with particular reference to seed ecology and seed banks. Perspect. Plant Biol. Evol. Syst..

[20] Schütz W (2000). Ecology of seed dormancy and germination in sedge (*Carex*). Perspect. Plant Ecol. Evol. Syst..

[21] van der Valk AG, Bremolm TL, Gordon E (1999). The restoration of sedge meadows: seed viability, seed germination requirements and seedling growth of *Carex* species. Wetlands.

[22] Mulhouse JM, Galatowitsch SM (2003). Revegetation of prairie pothole wetlands in the mid-continental us: twelve years post-reflooding. Plant Ecol..

[23] Yetka LA, Galatowitsch SM (1999). Factors affecting revegetation of *Carex lacustris* and *Carex stricta* from rhizomes. Restor. Ecol..

[24] Zhang DJ, Wang XH, Tong SZ (2016). Plant diversity in the restored riparian wetlands along the downstream of Songhua River. Wetland Sci..

[25] Wang GD, Wang M, Yuan YX, Lu XG, Jiang M (2014). Effects of sediment load on the seed bank and vegetation of *Calamagrostis angustifolia* wetland community in the National Natural Wetland Reserve of Lake Xingkai, China. Ecol. Eng..

[26] Wang GD, Jiang M, Lu XG, Wang M (2013). Effects of sediment load and water depth on the seed banks of three plant communities in the National Natural Wetland Reserve of Lake Xingkai, China. Aqua. Bot..

[27] Baskin, C. C. & Baskin, J. M., *Seeds. Ecology, biogeography, and evolution of dormancy and germination*. Academic Press, San Diego, California (2001).

[28] Wilcox DA, Kowalski KP, Hoare HL, Carlson ML, Morgan HN (2008). Cattail invasion of sedge/grass meadows in Lake Ontario: photointerpretation analysis of sixteen wetlands over five decades. J. Great Lakes Res..

[29] Meng M (2013). Wetland protection and restoration in Sanjing Plain and its implications for practice. Chinese Nat..

[30] Lishawa SC, Albert DA, Tuchman NC (2010). Water level decline promotes *Typha* X *glauca* establishment and vegetation change in Great Lakes coastal wetlands. Wetlands.

[31] Woo I, Zedler JB (2002). Can nutrients alone shift a sedge meadow towards dominance by the invasive *Typha*×*glauca*?. Wetlands.

[32] Chen H, Zamorano MF, Ivanoff D (2010). Effect of flooding depth on growth, biomass, photosynthesis, and chlorophyll fluorescence of *Typha domingensis*. Wetlands.

[33] Boers AM, Veltman RL, Zedler JB (2007). *Typha*×*glauca* dominance and extended hydroperiod constrain restoration of wetland diversity. Ecol. Eng..

[34] Xue ZS, Jiang M, Lu XG, Liu XH, Zhao DY (2012). Influence of agricultural exploitation on ecosystem services: a case study on middle and lower reaches of Nongjiang River and Bielahong River in the Sanjiang Plain. Wetland Sci..

[35] Yi, F. K., Yi, X. Y., Lou, Y. J. & Wang, X. *Wetland Wild Vascular Plants in Northeastern China*. Science Press, Beijing, China (2008).

[36] Fu, P. Y. *Clavis Plantatrum Chinae Boreali-Orientalis*. Science Press, Beijing. China (1995).

