# Supplementary material for: Duration of farming is an indicator of natural restoration potential of sedge meadows
Source: Sci Rep. 2017 Sep 6;7:10692. doi: 10.1038/s41598-017-11429-0 (PMC5587675; doi:10.1038/s41598-017-11429-0)
Supplement: Supplementary file 1 — Supplementary Table S1 [file 41598_2017_11429_MOESM1_ESM.pdf]

Supplementary Information

Duration of farming is an indicator of natural restoration potential of sedge meadows

Guodong Wang [1]*, Ming Wang [2], Xianguo Lu [1] & Ming Jiang [1]*

[1] Key Laboratory of Wetland Ecology and Environment, Northeast Institute of Geography and Agroecology, Chinese Academy of Sciences, Changchun, Jilin, 130102, China; [2] Institute for Peat and Mire Research, Northeast Normal University, Changchun, Jilin, 130024,China

*Corresponding author: G.W. (wanggd@iga.ac.cn); M.J. (jiangm@iga.ac.cn).

Table S1

Species list in this study and seed density per pot (mean±SE) of each species germinating in soils from natural sedge meadows, soybean fields farmed for various lengths of time in the Sanjiang Plain, northeastern China.

		Mean density	
species	category	Sedge meadows	Farm fields
**Drained**			
Achillea millefolium L. [p]	Wetland	0.02±0.013	0±0
Agrostis clavata Trin. [a]	Wetland	0.85±0.37	0.01±0.009
A. macranthera Chang. et Skv. [p]	Wetland	0.59±0.205	0.36±0.087
Alisma orientale (Sam.) Juz. [p]	Wetland	0.01±0.009	0.19±0.122
Alopecurus pratensis L. [p]	Wetland	0.58±0.129	0.49±0.139
Ambrosia artemisifolia L. [a]	Non-wetland	0±0	0.01±0.009
Androsace filiformis Retz. [a]	Wetland	0.39±0.117	0.04±0.036
Anemone dichotoma L. [p]	Wetland	0.22±0.103	0.02±0.017
Artemisia stolonifera (Maxim.) Kom. [p]	Non-wetland	0±0	0.72±0.307
A. aurata Komar. [a]	Non-wetland	0±0	0.02±0.013
A. japonica Thunb. [p]	Non-wetland	0.03±0.015	0.08±0.035
A. integrifolia L. [p]	Wetland	0.35±0.103	0.02±0.012
A. carvifolia Buch.-Ham. [a]	Non-wetland	0±0	0.25±0.101
Arthraxon hispidus (Thunb.) Makino. [a]	Non-wetland	0±0	0.11±0.038
Beckmannia syzigachne (Steud.) Fern. [a]	Wetland	0.65±0.207	0.27±0.083
Betula fruticosa Pall. [S]	Wetland	0.5±0.115	0±0
Bidens bipinnata L. [a]	Wetland	0.01±0.009	0.03±0.015
Calamagrostis angustifolia Kom. [p]	Wetland	7.47±0.778	2.1±0.301
C. langsdorffii (Link.) Trin. [p]	Wetland	0.51±0.079	0±0
Callitriche palustris L. [a]	Wetland	0.3±0.127	0.13±0.065
Caltha palustris L. var. *sibirica* Regel. [p]	Wetland	0.21±0.08	0.02±0.017
Capsella bursa-pastoris (Linn.) Medic. [a]	Non-wetland	0±0	0.14±0.036
Carex humida Y. L. Chang. et Y. L. Yang. [p]	Wetland	0.69±0.194	0.13±0.052

C. lasiocarpa Ehrh. [p]	Wetland	0.92±0.179	0.14±0.073
C. limosa L. [p]	Wetland	0.21±0.099	0.06±0.06
C. orthostachys C.A. Mey. [p]	Wetland	0.53±0.194	0.11±0.095
C. pseudo-curaica Fr. Schmidt. [p]	Wetland	0.43±0.179	0.12±0.11
C. meyeriana Künth. [p]*	Wetland	0.57±0.154	0±0
C. appendiculata (Trautv.) Kükenth. [p]*	Wetland	0.38±0.167	0±0
C. schmidtii Meinsh. [p]*	Wetland	0.31±0.189	0.02±0.01
Centipeda minima (L.) A. Br. et Aschers. [a]	Wetland	0.16±0.062	0.03±0.019
Chenopodium glaucum L. [a]	Non-wetland	0.12±0.042	1.05±0.278
Cirsium maackii Maxim. [p]	Wetland	0.11±0.033	0.04±0.019
Commelina communis L. [a]	Wetland	0±0	0.01±0.009
Comarum palustre L. [p]	Wetland	0.49±0.181	0.24±0.08
Cyperus difformis L. [a]	Wetland	0.01±0.009	0.02±0.012
C. exaltatus Retz. [p]	Wetland	0.02±0.013	0±0
C. fuscus L. [a]	Wetland	0.02±0.019	0±0
C. glomeratus L. [a]	Wetland	0.07±0.029	0±0
Digitaria sanguinalis (L.) Scop. [a]	Non-wetland	0±0	0.14±0.064
Echinochloa crusgalli (L.) Beauv. [a]	Wetland	0.17±0.064	0.9±0.146
Eleocharis mamillata Lindb.f. [p]	Wetland	0.14±0.009	0.03±0.019
E. ovata (Roth.) Röem. et Schult. [a]	Wetland	0.34±0.069	0±0
Epilobium palustre L. [p]	Wetland	0.09±0.039	0±0
Erigeron acer L. [a]	Non-wetland	0.08±0.045	0.41±0.097
Euphorbia humifusa Willd. [a]	Non-wetland	0±0	0.01±0.009
Galium trifidum L. [p]	Wetland	0.57±0.185	0.12±0.046
G. manshuricum Kitag. [p]	Wetland	0.11±0.009	0±0
Geranium vlassowianum Fisch. Ex Link. [p]	Wetland	0.03±0.016	0±0
Glyceria spiculosa (Fr.Schmidt.) Rosh. [p]	Wetland	1.81±0.485	0.05±0.037
Glycine soja Sieb. et Zucc. [a]	Non-wetland	0.01±0.009	0±0
Gnaphalium mandshuricum Kirp. [a]	Wetland	1.67±0.48	1.83±0.562

Hieracium hololeion Maxim. [p]	Wetland	0.05±0.024	0.01±0.009
Hypericum ascyron L. [p]	Wetland	0.29±0.084	0.08±0.031
Inula japonica Thunb. [p]	Wetland	0.05±0.031	0±0
Iris laevigata Fisch.et C. A. Mey. [p]	Wetland	0.02±0.013	0±0
Juncellus serotinus (Rottb.) C. B. Clarke. [p]	Wetland	0.64±0.148	0.03±0.027
*Juncus papillosus*Franch. et Sav. [p]	Wetland	1.46±0.394	0.26±0.111
J. effusus L. [p]	Wetland	0.26±0.077	0.09±0.04
J. wallichianus Laharpe. [p]	Wetland	0.21±0.106	0±0
Lathyrus palustris L. var. pilosus (Cham.) Ledeb. [p]	Wetland	0.47±0.231	0±0
L. quinquenervius (Miq.) Litv. Ex Kom. Et Alis. [p]	Wetland	0.06±0.012	0+0
Lindernia procumbens (Krock.) Bobas. [a]	Wetland	0.24±0.127	0.58±0.25
Lycopus lucidus Turcz. [p]	Wetland	0.24±0.071	0.01±0.009
Lysimachia thyrsiflora L. [p]	Wetland	0.11±0.034	0±0
Lythrum salicaria L. [p]	Wetland	0.05±0.009	0±0
Monochoria vaginalis (Burm. f.) Presl. [a]	Wetland	0±0	0.02±0.017
Murdannia keisak (Hassk.) Hand-Mazz. [a]	Wetland	0.27±0.102	0±0
Pennisetum alopecuroides (L.) Spreng. [a]	Non-wetland	0±0	0.01±0.009
Plantago asiatica L. [p]	Wetland	0.37±0.096	0.06±0.026
Poa subfastigiata Trin. [p]	Wetland	0.53±0.134	0.12±0.057
P. Palustris L. [p]	Wetland	0.31±0.154	0.03±0.019
Polygonum amphibium L. [p]	Wetland	1.83±0.486	1.77±0.86
P. aviculare L. [a]	Wetland	0.07±0.052	0±0
P. hydroplper L. [a]	Wetland	0.03±0.021	0.02±0.012
P. korshinskianum Nakai. [a]	Wetland	0.1±0.053	0.03±0.019
P. persicaria L. [a]	Wetland	0.79±0.21	0.18±0.056
Pycreus sanguinolentus (Vahl) Nees. [a]	Wetland	0.42±0.173	0.22±0.079
P. korshinskyl (Meinsh.) V. Krecz. [a]	Wetland	0.08±0.033	0.03±0.026
Rorippa palustris (Leyss.) Bess.[bp]	Wetland	1.21±0.338	1.92±0.999
Rumex patientia L. var. *callosus* Fr. Schmidt[p]	Non-wetland	0.15±0.078	0.29±0.118

Sagittaria trifolia L. [p]	Wetland	0.41±0.076	0.67±0.19
S. natans Pall. [p]	Wetland	0.12±0.043	0±0
Salix rosmarinifolia L. [s]	Wetland	1.09±0.12	0.37±0.081
S. myrtilloides L. [s]	Wetland	0.18±0.039	0.14±0.039
S. floderusii Nakai. [s]	Wetland	0.07±0.027	0.06±0.022
Sanguisorba parviflora (Maxim.) Takeda. [p]	Wetland	0.07±0.032	0±0
Saussurea amurensis Turcz. Ex DC. [p]	Wetland	0.11±0.041	0.01±0.009
Scirpus fluviatilis (Torrey.) A.Gray. [p]	Wetland	0.17±0.06	0.07±0.027
Scutellaria regeliana Nakai. [p]	Wetland	0.02±0.009	0±0
Setaria viridis (L.) Beauv. [a]	Non-wetland	0±0	0.06±0.026
Sium suave Walt. [p]	Wetland	0.09±0.041	0±0
Spiraea salicifolia L. [s]	Wetland	0.11±0.02	0.01±0.009
Stachys baicalensis Fisch.Ex Benth. [p]	Wetland	0.35±0.087	0.08±0.045
S. japonica Miq. [p]	Wetland	0.06±0.022	0.02±0.012
Stellaria filicaulis Makino. [p]	Wetland	0.65±0.335	0±0
S. longifolia Muehl. [p]	Wetland	1.35±0.61	0±0
S. media (L.) Cyr. [ab]	Wetland	0.01±0.009	0.01±0.009
S. neglecta Weihe. [ab]	Wetland	0.05±0.028	0.03±0.019
S. radians L. [p]	Wetland	0.03±0.021	0.02±0.017
Sonchus brachyotus DC. [p]	Wetland	0.41±0.064	0.11±0.041
Thalictrum squarrosum Steph. Ex Willd. [p]	Non-wetland	0±0	0.02±0.013
Triglochin palustre L. [p]	Wetland	0.05±0.031	0.01±0.009
Typha angustifolia L. [p]	Wetland	0.18±0.085	0.87±0.103
Viola patrinii DC. Ex Ging. [p]	Wetland	0.14±0.049	0±0
**Flooded**			
Alisma orientale (Sam.) Juz. [p]	Wetland	0.342±0.124	1.421±0.72
Callitriche palustris L. [a]	Wetland	0.142±0.069	0.248±0.081
Ceratophyllum demersum L. [p]	Wetland	1.49±0.45	0±0
Potamogeton crispus L. [p]	Wetland	2.58±1.78	0.733±0.141

P. malaianus Miq. [p]	Wetland	1.74±0.42	0.688±0.265
P. perfoliatus L. [p]	Wetland	0.206±0.173	0±0
Sagittaria trifolia L. [p]	Wetland	0.87±0.21	2.44±0.87
Sium suave Walt. [p]	Wetland	1.06±0.21	0±0
Typha angustifolia L. [p]	Wetland	0.47±0.27	2.99±0.41
Vallisneria spiralis L. [p]	Wetland	1.06±0.998	0.65±0.162

[a] annual, [b] biennial, [p] perennial, [s] shrub; [*] tussock-forming sedge species.
